# Nanostructured materials with biomimetic recognition abilities for chemical sensing

**DOI:** 10.1186/1556-276X-7-328

**Published:** 2012-06-21

**Authors:** Sadia Zafar Bajwa, Ghulam Mustafa, Renata Samardzic, Thipvaree Wangchareansak, Peter A Lieberzeit

**Affiliations:** 1 Department of Analytical Chemistry, University of Vienna, Währinger Strasse 38, Vienna, 1090, Austria; 2 Department of Chemistry, Faculty of Science, Kasetsart University, Chatuchak, Bangkok, 10900, Thailand

**Keywords:** Bivalent copper ions, Organic vapors, Wheat germ agglutinin lectin, *Escherichia coli*, Quartz crystal microbalance

## Abstract

Binding features found in biological systems can be implemented into man-made materials to design nanostructured artificial receptor matrices which are suitable, e.g., for chemical sensing applications. A range of different non-covalent interactions can be utilized based on the chemical properties of the respective analyte. One example is the formation of coordinative bonds between a polymerizable ligand (e.g., *N*-vinyl-2-pyrrolidone) and a metal ion (e.g., Cu(II)). Optimized molecularly imprinted sensor layers lead to selectivity factors of at least 2 compared to other bivalent ions. In the same way, H-bonds can be utilized for such sensing purposes, as shown in the case of *Escherichia coli*. The respective molecularly imprinted polymer leads to the selectivity factor of more than 5 between the W and B strains, respectively. Furthermore, nanoparticles with optimized Pearson hardness allow for designing sensors to detect organic thiols in air. The ‘harder’ MoS_2_ yields only about 40% of the signals towards octane thiol as compared to the ‘softer’ Cu_2_S. However, both materials strongly prefer molecules with -SH functionality over others, such as hydrocarbon chains. Finally, selectivity studies with wheat germ agglutinin (WGA) reveal that artificial receptors yield selectivities between WGA and bovine serum albumin that are only about a factor of 2 which is smaller than natural ligands.

## Background

Recent focus of material scientists and engineers is to design economically and functionally efficient receptors allowing for substrate binding characteristics similar to those of natural systems. Highly selective recognition is one of the fundamental features of life-governing biological functions, including immune reactions and catabolic and anabolic enzymatic processes to name the most important ones. Despite optimal selectivity, there are some inherent limitations of these natural systems, such as degradation in harsh environments and limited life time and stability. Furthermore, especially antibodies bind to their antigens very strongly, thus limiting reversibility of the recognition process. However, the non-covalent interaction networks governing biological systems can be mimicked to produce nanostructured artificial receptors with extended operation time and shelf life [1-4]. Various types of analyte-receptor interactions can be implemented in this way, such as affinity-based ones, hydrophobic interactions, hydrogen bonds, and polar interactions. In the case of ‘pure’ affinity interactions with the outer surface of the artificial receptor, nanostructuring the recognition layer should lead to substantially enhanced sensor responses due to the larger internal surface.

If both steric and geometric features are to be addressed, molecular imprinting [5] via covalent [6], non-covalent [7,8] or semi-covalent approaches [9], respectively, can be implemented. Obviously, the interactions between the analyte and matrix play a key role in controlling the recognition ability of the material in any case. Thus, rationally functionalized materials, be these imprinted by the analyte of interest itself or possess affinity towards it, are able to distinguish the template from interfering species even when having related geometrical and chemical structures. Such (nano)materials constitute very promising artificial receptors for the detection of a variety of analytes from the subnano- to microlevel, such as ions [10], volatile organic compounds [11], cells, yeasts [12], viruses [13], and proteins [14] to name a few. The present work summarizes some aspects of interactions leading to the design of useful chemical sensors including affinity nanoparticles, generating the molecularly imprinted polymer (MIP) with the smallest possible templates and aspects of comparing artificial and natural receptors, respectively. The recognition abilities of the prepared materials are assessed by applying them onto quartz crystal microbalance (QCM) transducers and by tracing the subsequent frequency shifts following analyte exposure.

## Methods

### Materials and chemicals

All chemicals were purchased either from Merck (Vienna, Austria), Sigma-Aldrich (Vienna, Austria) or Alfa Aesar (Karlsruhe, Germany) as analytical grade or the highest available synthetic grade and were utilized without further purification. AT-cut quartz crystal sheets (10 MHz fundamental frequency) and the gold paste (for the screen printing of electrode over QCM sheets) were purchased from Great Microtama Electronics (Surabaya, Indonesia) and HERAEUS (Hanau, Germany), respectively.

### Synthesis of cu(II)-imprinted polymer

Three milligrams of CuCl_2_·2H_2_O was dissolved in 300 mg of *N*-vinyl-2-pyrrolidone (NVP). Thirty milligrams of this Cu(II)-NVP complex was then mixed with 60 mg of ethylene glycol dimethylacrylate, 3 mg of 2,2′-azo-bis-isobutyronitrile, and 500 μl acetonitrile. This prepolymer mixture was kept under UV for 45 min to polymerize. Finally, 5 μl of this solution was drop-coated over one electrode pair of the QCM to achieve layer heights of about 400 nm that were verified by network analyzer measurements. A reference polymer was prepared and coated in exactly the same manner excluding the addition of copper ions. Distilled water was used to extract the metal from the imprinted polymer layer to obtain recognition sites.

### Synthesis of molybdenum disulphide nanoparticles

Molybdenum disulphide (MoS_2_) nanoparticles (NPs) were prepared from Mo(CO)_6_ and elemental sulfur following an already published synthesis protocol [15,16]. These papers also discuss the ways leading to the optimized sensor materials.

### Synthesis of copper sulfide nanoparticles

Copper sulfide (Cu_x_S) particles were synthesized via a photochemical method [17]: 5 ml of CuSO_4_ (1 mol/l) was mixed drop wise with 15 ml Na_2_S_2_O_3_ (1 mol/l), resulting in a bright green solution. This was kept at ambient conditions for a week without stirring. Then, black green nanoparticles were collected from the solution by centrifugation and washed several times with distilled water to remove unwanted byproduct and impurities. Finally, particles were dried at 60°C overnight.

### Synthesis of polymer for surface imprinting of WGA lectin

The procedure adopted for preparing optimized surface imprints of wheat germ agglutinin (WGA) lectin has been described earlier [18].

### Synthesis of *Escherichia coli* surface MIP

Polyurethane (PU) matrix was prepared by first dissolving 50.4 mg of poly(4-vinylphenol) and 4.3 mg of phloroglucinol in tetrahydrofuran (THF) and then adding 56.38 mg of diphenylmethan-4,4′-diisocyanat. The reaction mixture was kept at room temperature for 20 min to complete the polymerization process until gel point was reached. One part of this stock prepolymer solution was diluted with 15 parts of THF and coated to obtain a polyurethane layer of 350 to 400 nm for the surface imprinting of *E. coli*. A glass stamp of *E. coli* was prepared and pressed against the PU oligomer on the QCM electrode. A 0.1% sodium dodecyl sulfate solution was used to wash out the *E. coli* and to obtain empty cavities.

### Atomic force microscopic characterization

The Veeco Nanoscope IVa AFM/STM system (Plainview, NY, USA) was employed for characterizing Cu_2_S, MoS_2_ nanoparticles, and surface imprints of *E. coli*. To acquire scans, silicon nitride atomic force microscopy (AFM) probes (model NP-S10 from Veeco) having cantilever spring constants of 0.58 to 0.06 N/m were used. All scans were recorded at ambient temperature (24 ± 2°C) in air via contact mode. Three samples of each material were synthesized and imaged at three distinct areas using the same tip. Among these images, one representative image is displayed. Data analysis for obtaining further information on the systems was carried out with the Nanoscope AFM system software shipped together with the Veeco system, while *E. coli*-MIP cavities are depicted using the WSxM free software by Nanotec Electrónica (Madrid, Spain)

## Results and discussion

**Figure 1:**
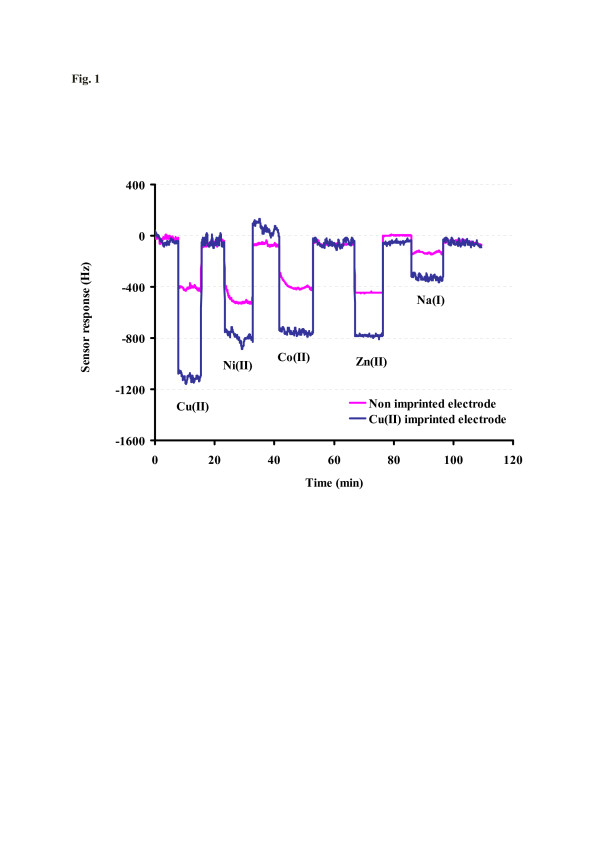
**QCM sensor response of Cu(II) MIP towards competing bivalent ions.** Layer height, 400 nm; concentrations, 1 mM each.

Metal ions as templates in molecular imprinting are interesting for two reasons: first, they are the smallest possible analytes in chemistry; second, they undergo directed interactions via coordinative bonds to a ligand. Therefore, we prepared imprinted polymers containing NVP as a functional monomer. First, experiments (results not shown here) indicated that the imprinting efficiency does not depend on the exact template, i.e., the respective anion only plays a marginal role. In fact, Cu(II) is able to form coordination complexes with NVP that are stabilized by the respective counter ion. Overall, the self-assembly procedures during imprinting thus indeed focus on the cation. Figure 1 shows the QCM frequency pattern for copper MIP and the respective reference material towards 1 mM aqueous solutions of copper and some interfering ions. Notably, the polymer preferentially adsorbs cupric ions as the mass effect for Cu(II) is at least two times larger than that for Ni(II), Zn(II), and Co(II), respectively. In all cases, the sensors were exposed to solutions containing only one ion species. Furthermore, the reference material is insensitive to the nature of the metal that justifies the absence of any recognition source in its structure which in turn means that the preformed coordination complexes during MIP synthesis indeed lead to selective recognition. This also explains the selectivity between the bivalent ions despite the comparably small difference in ionic sizes as radii for Cu(II), Ni(II), Co(II), and Zn(II) ions are 87, 83, 79, and 88 pm, respectively [19]. Na(I), on the other hand, is not only much larger in diameter (160 pm), but also does not form complexes. Therefore, it can be inferred that Cu(II) binds to NVP, and the structure and geometry of the complex are determined by the coordination sphere of the copper ion center. This also explains the substantial selectivity occurring despite the fact that the noble metal ions all have a rather similar globular geometry. Of course, further detailed studies are necessary for this system to further elucidate the recognition processes. Sensor responses on the non-imprinted material seem to be substantial. However, they are basically the same for all bivalent ions (and much smaller for Na(I)). This is a strong indication that the frequency shift in this case does not only depend on the mass change, but is also substantially determined by the change in conductivity between the deionized water used as the background matrix and the respective ionic solutions.

**Figure 2:**
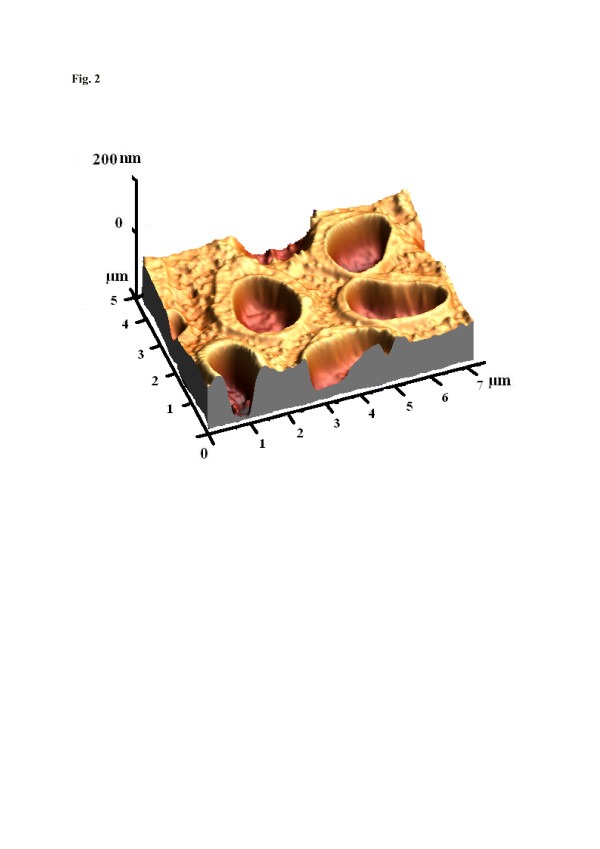
**AFM image of*E. coli*W strain imprints.** The imprints are 1.5 to 2.5 μm large and about 100 nm deep, casted on the polyurethane substrate; scan scale, 7 × 5 μm; scan rate, 0.897 Hz.

**Figure 3:**
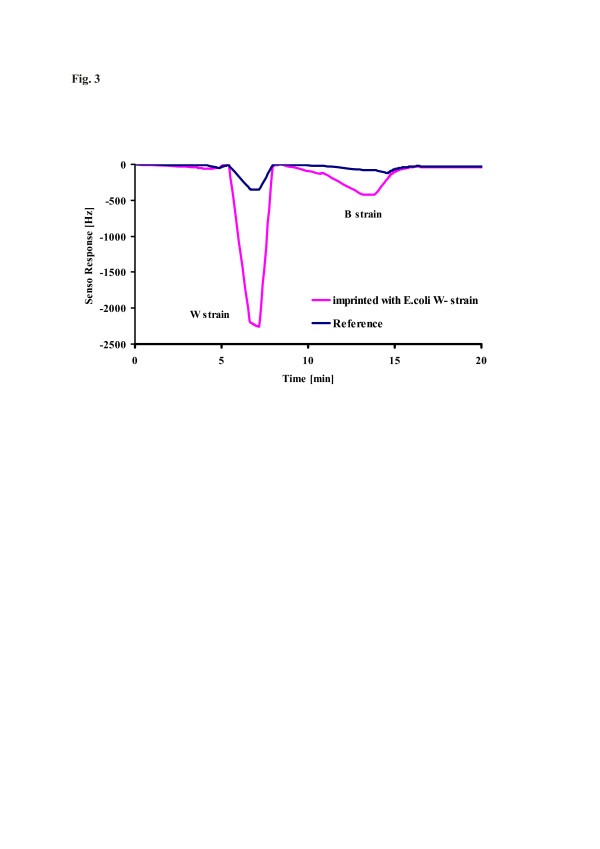
**Substrate selectivity of*E. coli*W strain-MIP towards 4 × 10**^**4**^**cells/ml suspensions of W and B strains.**

Not only coordinative bonds are highly directed, but also hydrogen bonds. They, of course, can also be applied to design materials with controlled binding networks for the selective detection of microorganisms. As an example for successful imprinting, *E. coli* has to be mentioned. It is a common, gram-negative, rod-shaped bacterium that is found in the intestines of warm-blooded animals. Surface imprinting, e.g., into a polyurethane matrix, leads to the respective surface cavities, as can be seen in Figure 2. It shows the AFM image of the polyurethane, where template cavities of *E. coli* (W strain) can be clearly identified, thus in principle, confirming the validity of the templating approach. Further insight into the material structure can be obtained from the sensor responses summarized in Figure 3. It shows the QCM data for both imprinted and non-imprinted materials towards 4 × 10^10^ cells/ml of the W and B strains of *E coli*, respectively. Clearly, the sensor responses reveal that the W strain is strongly preferred over the B strain in this case as its mass effect is four times larger. Additionally, bacteria of the W strain are usually slightly larger than those of the B strain. Therefore, cavities optimized for the larger species should also incorporate the smaller ones, meaning that also B strain bacteria should, in principle, be incorporated into cavities templated with the W strain specimen. The cell walls of *E. coli* consist of liposacchrides (LPS) of characteristic sequences. Bacteria are classified on the basis of these LPS and different types of antigen attached with the lipids [20]. In the case of MIP sensors, these surface sacchrides interact with the receptor material via the functional monomer, explaining the comparably high selectivity factor of 4.

**Figure 4:**
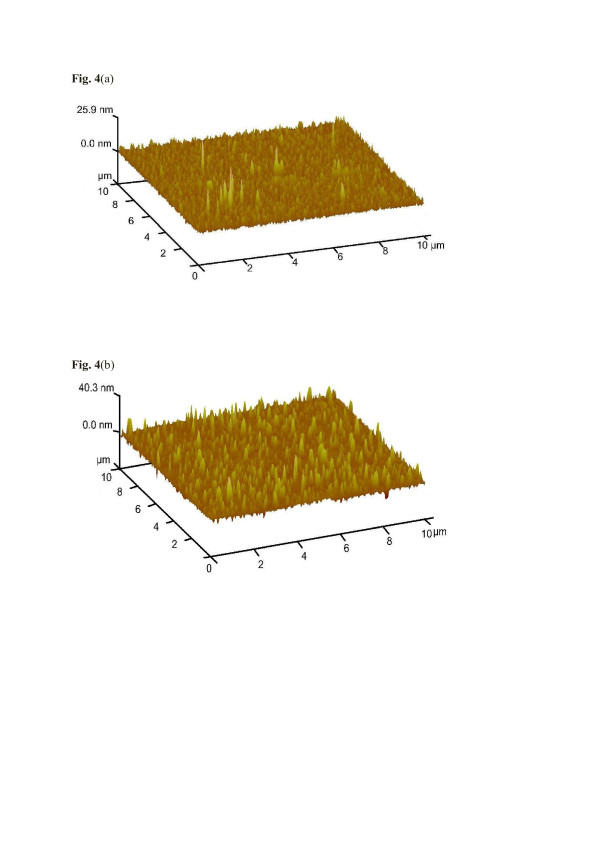
**AFM image of MoS**_**2**_**NPs and Cu**_**2**_**S NPs.** (**a**) AFM image of MoS_2_ NPs revealing that the size is about 50 to 100 nm; scan size, 10 × 10 μm; scan rate, 2.96 Hz. (**b**) AFM image of Cu_2_S NPs; the size is about 50 to 100 nm; scan size, 10 × 10 μm; scan rate, 1.25 Hz.

**Figure 5:**
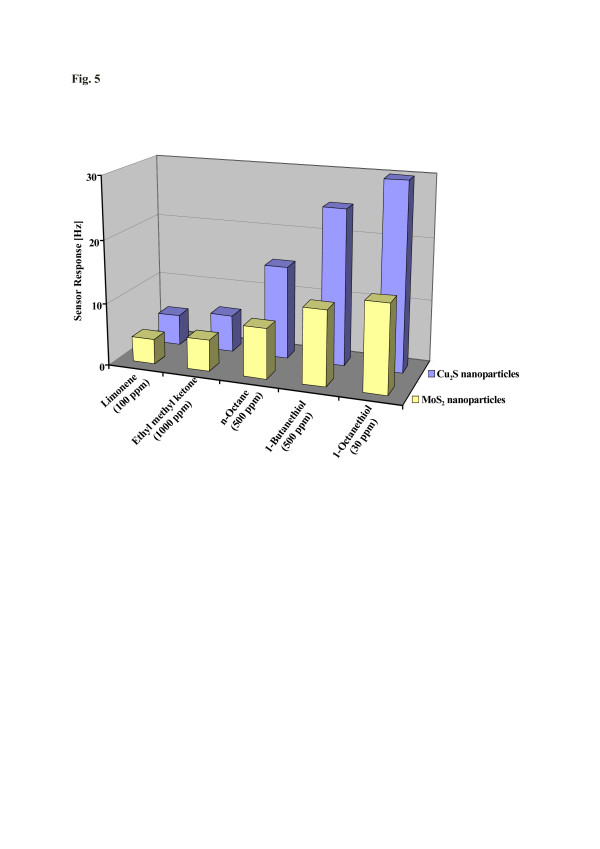
**MoS**_**2**_**and Cu**_**2**_**S NPs-QCM selectivity pattern towards different organic vapors.**

**Figure 6:**
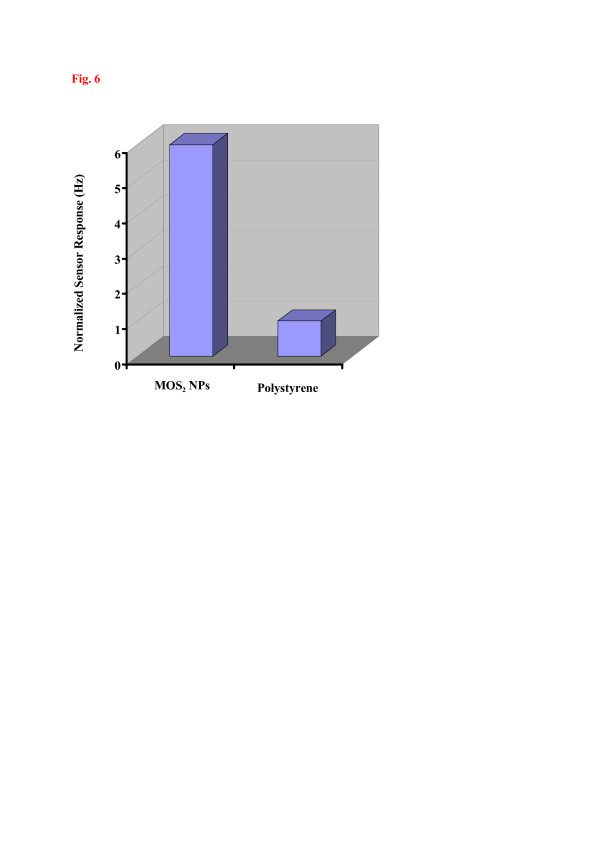
**Normalized sensor responses of MoS**_**2**_**NPs- and polystyrene-QCM towards 10 ppm 1-octanethiol vapors.**

Affinity interactions are further versatile means to achieve straightforward and robust chemical sensors [16], whose response is indirectly proportional to the diameter of the nanoparticles applied. Such affinity can be determined by a range of properties; one of which is the Pearson hardness scale. There, thiols are categorized as ‘soft’ so an appropriate metal substrate being also soft should lead to optimized affinity. To test this claim and to demonstrate this concept, MoS_2_ and Cu_2_S nanoparticles have been synthesized for sensing thiol vapors. Figure 4a,b displays the respective AFM images showing that both compounds can be synthesized in the shape of nanoparticles having 100 and 150 nm in diameter, respectively. The size distribution in both cases is appreciably small. Figure 5 summarizes the sensor data collected when MoS_2_ and Cu_2_S nanoparticles are exposed to different types of organic vapors. It can be seen clearly that both kinds of nanomaterials strongly prefer the thiol functionality over the other ones: the highest sensitivities are achieved for both 1-butanethiol and 1-octanethiol. For octane and other analytes, the responses are at least a factor of 2 (1-butanethiol) and 3 (1-octanethiol) lower, respectively. This selectivity pattern gives substantial evidence that the driving force behind recognition is the Pearson hardness. Furthermore, increasing the metal softness from Mo(IV) to Cu(I), the resulting frequency shifts towards both 1-butanethiol and 1-octanethiol become three times larger, thus strongly supporting the presence of such non-covalent interactions in this case. Additionally, Figure 6 shows the sensor signals of the MoS_2_ nanoparticles and polystyrene thin film, respectively, towards 10 ppm 1-octanethiol. The response of polystyrene is six times lower as compared to MoS_2_ nanoparticles. PS does not contain any SH-functionality, so the sensor responses give is strong evidence in support of the aforementioned hard-soft interactions.

**Figure 7:**
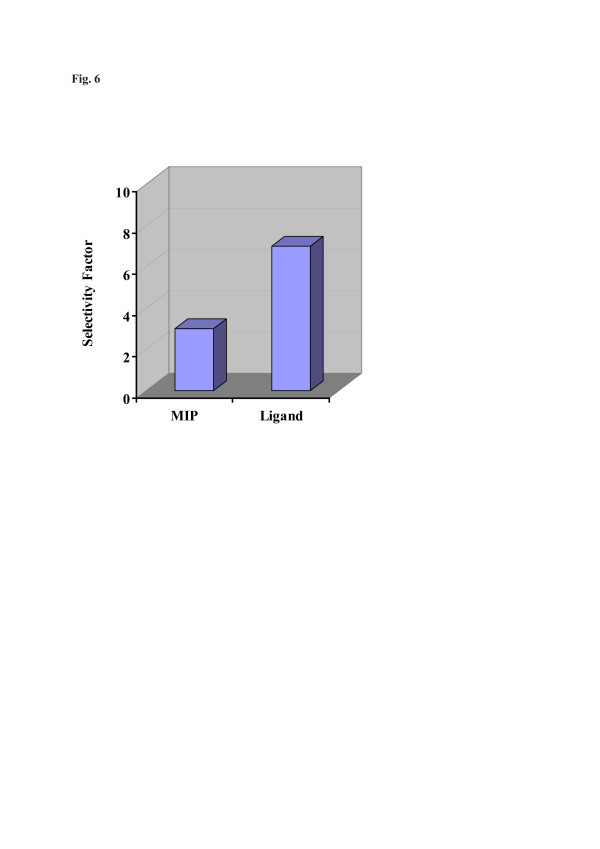
Selectivity factor when WGA-MIP and ligand-coated QCM are exposed towards WGA and BSA, respectively.

Finally, comparing artificial receptor materials with natural ones holds a key for assessing their recognition abilities for making biomimetic setups. One system, where this can be achieved in a rather straightforward way, is WGA lectin for which successful MIP has already been reported [18]. WGA lectin is a gylcoprotein, and its natural receptor is an oligosaccharide with a glucosamine moiety. The immobilization of ligand on the QCM surface also leads to sensor layers for selective and reversible binding of WGA lectin. Figure 7 summarizes the selectivities that can be achieved from both sensor layers, i.e., the ‘artificial receptor’ MIP and the ligand, when exposed to WGA and bovine serum albumin (BSA) solutions, respectively, with a concentration of 160 μg/l. BSA is a serum protein having a similar size with WGA [21]. Evidently, the MIP approach leads to selectivity factors of about 3, whereas the ligand system prefers WGA by a factor of more than 7. Although this is still more than a factor of 2 which is better than the MIP system, it still shows that fully artificial recognition systems already approach natural ones in terms of selectivity in some cases.

## Conclusion

Highly selective artificial recognition materials can be achieved by nanostructuring matrices and making use of a range of non-covalent intermolecular interactions, such as coordinative bonds, hydrogen bonds, and affinity interactions based on the Pearson hardness of both the analyte and the receptor layer. Both molecularly imprinted polymers and affinity material nanoparticles reach appreciable analyte-layer interactions, leading to measurable sensor signals in mass-sensitive detection by quartz crystal microbalances. This is true for both small and large species, as represented here by metal ions and bacteria, respectively. In the case of WGA lectin, the fully artificial imprinted system already yields selectivities of the same order of magnitude as can be achieved with immobilized ligand species of this protein.

## Competing interests

The authors declare that they have no competing interests

## Authors’ contributions

SZB has carried out the experiments for ion imprinting and contributed to the writing of the manuscript. GM was responsible for the affinity nanoparticles for thiol detection. RS focused on *E. coli* MIP and their evaluation. TW studied the WGA lectin system. PAL supervised all work and was responsible for the final manuscript version. All authors read and approved the final manuscript.

## Authors’ information

SZB currently pursues her Ph.D. studies at the University of Vienna funded by a scholarship of the Pakistani Higher Education Commission. She has specialized on generating highly selective MIP materials for ionic analytes.

GM obtained his Ph.D. from the University of Vienna in July 2011, where he studied on a scholarship by the Pakistani Higher Education Commission. He has specialized on gas sensing applications of inorganic nanoparticles.

RS currently pursues her Ph.D. studies at the University of Vienna. She has specialized on bacteria MIP and their application in real-life environments.

TW pursues her Ph.D. studies via the Thai Royal Golden Jubilee Grant Program in a joint project between Kasetsart University, Bangkok and the University of Vienna. Her research interests are in elucidating glycoside-protein interactions in natural systems.

PAL holds a professorship in Analytical Chemistry at the University of Vienna. He is a chemist having received his Ph.D. from the University of Vienna in 1999 and his postdoctoral lecture qualification in 2007. He is especially interested in developing chemical sensors for real-life environments including complex mixtures and applies pattern recognition methods for sensor arrays. This work to date has resulted in about 65 publications in peer-reviewed journals and approximately 80 conference contributions. Furthermore, he is strongly engaged in projects within the European Research Framework Programs.
